# The Influence of Psychotherapy on Peripheral Brain-Derived Neurotrophic Factor Concentration Levels and Gene Methylation Status: A Systematic Review

**DOI:** 10.3390/jcm10194424

**Published:** 2021-09-27

**Authors:** Michal Piotrkowicz, Marlena Janoska-Jazdzik, Tytus Koweszko, Agata Szulc

**Affiliations:** Department of Psychiatry, Faculty of Health Sciences, Medical University of Warsaw, Partyzantow 2/4, 05-802 Pruszkow, Poland; marlena.janoska@gmail.com (M.J.-J.); koweszko@gmail.com (T.K.); agataszulc@poczta.onet.pl (A.S.)

**Keywords:** psychotherapy, BDNF, brain-derived neurotrophic factor, methylation, epigenetics

## Abstract

Psychotherapy is a well-established method of treating many mental disorders. It has been proven that psychotherapy leads to structural and functional changes in the brain; however, knowledge about the molecular and cellular mechanisms of these changes is limited. Neuroplasticity and one of its mediators, brain-derived neurotrophic factor (BDNF), are potential research targets in this field. To define the role of BDNF concentration in serum, or in plasma, and BDNF promoter gene methylation in saliva or leucocytes, in psychotherapy, an extensive literature search was conducted in the PubMed and Web of Science databases. The literature review was conducted based on papers published up until May 2021 that included pre and post psychotherapy measurements of either BDNF concentration levels or promoter gene methylation status. Ten studies were indicated as eligible for analysis: eight studies that investigated peripheral BDNF concentration levels, one study that investigated methylation status, and one study that included an evaluation of both subject matters. Patients underwent cognitive behavioral therapy or interpersonal psychotherapy. Patients were diagnosed with borderline personality disorder, major depressive disorder, anorexia nervosa, bulimia nervosa, or post-traumatic stress disorder. There were only three of the nine studies that showed statistically significant increases in BDNF concentration levels after psychotherapy. The two studies that involved BDNF gene methylation status showed a decrease in methylation after dialectical behavioral therapy of borderline patients.

## 1. Introduction

Although our understanding of how psychotherapy works in terms of psychological mechanisms is improving, there is considerably less attention given to the underlying effects of the biological processes. Several studies using different neuroimaging techniques, have shown that structural and functional changes occur during the process of psychotherapy [1]. However, molecular mechanisms remain unclear. Brain-derived neurotrophic factor (BDNF) is the most studied member of the growth factor neurotrophic family, linked heavily to neuroplastic processes. It supports the survival, growth, and differentiation of neurons, as well as forming new synapses. Its synthesis is linked to neuronal activity. Due to its role in activity-dependent synaptic plasticity, BDNF plays a pivotal role in long-term modifications in synaptic transmission, caused by specific stimuli and their combinations. This process underlays memory and learning [2], and therefore BDNF is linked to those cognitive processes. During the course of aging, a decrease in peripheral BDNF concentration levels is linked to impaired cognitive performance [3,4,5]. The BDNF gene has a non-synonymous single nucleotide polymorphism (nsSNP). Substitution of valine by methionine is known to reduce the activity-regulated secretion of BDNF and is correlated with memory deficits in healthy individuals [6,7,8]. BDNF is also involved in the pathogenesis of neuropsychiatric disorders, such as schizophrenia, major depressive disorder (MDD), bipolar disorder, anxiety, and eating disorders [9]. People with the G to A allele (Val66Met) of the rs6265 polymorphism have a higher risk of depression [10], and it is also strongly linked to bipolar disorder [11] and suicidal behavior [12]. BDNF concentration levels in blood are decreased in depression, bipolar disorder, schizophrenia, anorexia nervosa, and Alzheimer’s disease [9,13,14,15,16]. Conversely, increased BDNF concentration levels are observed in epilepsy; BDNF with its excitatory properties has a pro-epileptogenic effect [17]. It is also known that early maltreatment produces persistent changes in methylation of BDNF promoter genes in rats and humans, leading to its decreased activity [18,19,20]. Increased methylation of BDNF promoter genes has been observed in patients with borderline personality disorder [21] and post-traumatic stress disorder [22]. This observation is not surprising as the patients have often experienced maltreatment during their childhood, and it may be especially important, as BDNF plays a role in moderating fear and stress responses, thus, enabling correct trauma processing [23]. Despite the evidence of increased BDNF methylation in PTSD, the results of studies exploring BDNF concentration levels in the blood of these patients are mixed. Some studies have shown increased concentration levels [24,25,26], while other studies have reported decreased concentration levels [27,28]. Nevertheless, the studies have shown an important function of BDNF in PTSD. Another environmental factor increasing methylation is prenatal exposure to tobacco [29] and acute stress, at least in animal models [19]. Alterations in BDNF promoter gene methylation are also observed in schizophrenia, depression, bipolar disorder, as well as in the brain tissue of suicide victims [30]. Treatment is not without an influence on BDNF. Long-term antidepressant treatment normalizes BDNF serum levels [14]. The substances studied were mainly selective serotonin reuptake inhibitors, serotonin and noradrenaline reuptake inhibitors, and tricyclic antidepressants [14,31,32,33]. Molendijk et al. [34] also showed, in their cross-sectional study, that untreated MDD patients had lower BDNF concentration levels than unremitted patients treated with antidepressants and patients in remission [34]. There are also suggestions that fluoxetine may increase BDNF concentration levels and improve cognitive functioning in vascular dementia [35], which may be due to upregulation of BDNF expression through stimulation of the serotonergic system [9]. Some studies have suggested that antidepressants bind to the BDNF receptor tropomyosin receptor kinase B (TrkB), which, apart from other effects, leads to the upregulation of BDNF expression [36,37]. Electroconvulsive therapy increases BDNF levels in depressed patients [38,39]. BDNF levels in plasma are consistently elevated after antipsychotic treatment with no link to patients’ responses to medication [15]. In a recent meta-analysis, Gomutbutra et al. [40] concluded that both exercise and meditation-based mindfulness interventions increased peripheral concentration levels of BDNF; however, in this case, the mechanism was unclear [40]. Furthermore, physical activity is known to positively influence BDNF levels [41], increasing its expression, probably through epigenetic modification [42,43]. The change of BDNF concentration during individual psychotherapy was recently studied in a review by Claudino et al. [44]; however, the review excluded studies in which group psychotherapy was used, as well as studies in which methylation of BDNF promoter gene was measured. The latter seems important, as disentangling the direct mechanism of action may be essential to clinical practice, for example, providing clues to combining multiple therapeutic approaches. In this systematic review, we address the relationship between psychotherapy and changes in peripheral BDNF levels, as well as epigenetic modification of BDNF genes, and therefore assess the importance of neuroplasticity as a mechanism of action of both group and individual psychotherapy.

## 2. Methods

### 2.1. Literature Search

**Figure 1:**
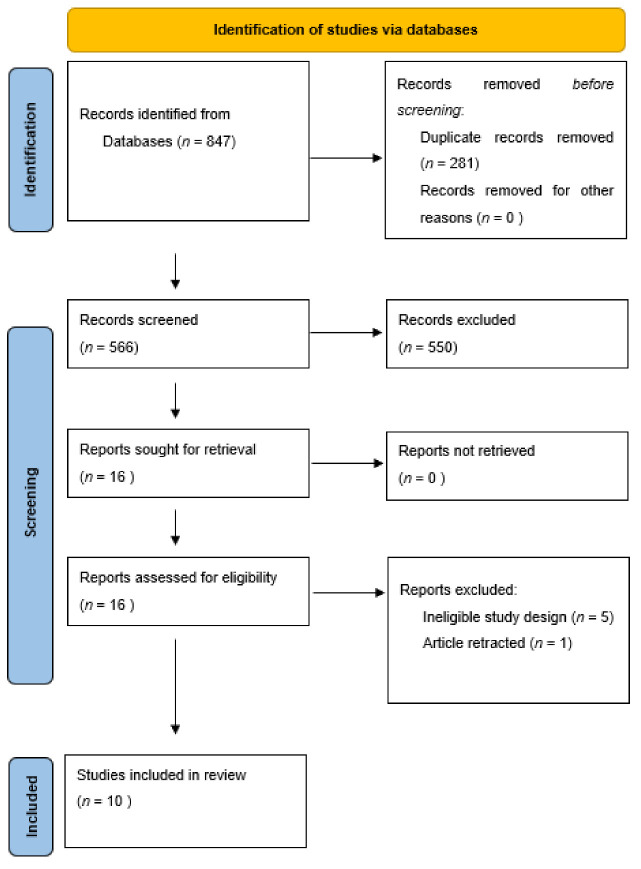
Flow diagram.

The literature search was conducted in the PubMed and Web of Science databases up until May 2021. The keywords “psychotherapy” and “BDNF” were used. Two researchers reviewed the databases independently and identified relevant abstracts. Afterwards, the eligibility of chosen papers was evaluated based on full text, and a second selection was performed. Studies that did not fulfill the inclusion criteria were discarded. In addition, manual searches in articles’ references that were deemed eligible for review, were conducted. All disagreements were resolved through discussion and reaching a consensus. The Preferred Reporting Items for Systematic Reviews and Meta-Analysis guidelines were followed [13]. Figure 1 represents the flow diagram summarizing screening process.

### 2.2. Inclusion Criteria

The inclusion criteria were defined as follows: (1) any original paper appearing in a peer-reviewed journal and (2) any longitudinal study that recruited patients with mental disorders and evaluated BDNF serum levels or BDNF gene methylation status before and after psychotherapy. Individual, group, and mixed psychotherapies were included.

### 2.3. Data Extraction and Variables Investigated

Two researchers independently extracted the following data: diagnosis, sample size, the use of a control group, type and duration of psychotherapy, main findings. The data were processed independently by two researchers and is summarized in Table 1.

### 2.4. Data Synthesis

The studies that fulfilled the inclusion criteria were reviewed in detail. A separate summary was achieved for peripheral BDNF concentration levels and methylation status.

### 2.5. Studies Retrieved

Ten studies were selected to be eligible for analysis. Nine of the studies focused on the change of BDNF protein in either serum or plasma. Two studies were identified that investigated BDNF promoter gene methylation. In the majority of studies, patients underwent some variant of cognitive-behavioral therapy (CBT), with the exception of two patients for whom interpersonal psychotherapy (IPT) was used. Patients were diagnosed with borderline personality disorder (BPD), major depressive disorder (MDD), anorexia nervosa (AN), bulimia nervosa (BN), or post-traumatic stress disorder (PTSD). The psychotherapy modalities used and the patient diagnoses are summarized in Table 1.

## 3. Results

### 3.1. Peripheral BDNF Concentration Levels

Three studies out of nine showed statistically significant growth of BDNF levels after psychotherapy; two of the studies investigated patients with eating disorders treated with a behavioral program with elements of cognitive therapy.

Yamada et al. [46] assessed BN patients (*N* = 7), who, after a four-week behavioral program and cognitive treatment, showed increased plasma BDNF concentration levels, without changes in BMI or scales measuring depressive and anxiety symptoms. However, a decrease in the frequency of binge eating and purging behaviors was observed.

Zwipp et al. [48] investigated underweight patients with AN (*N* = 14), who had an increase in serum BDNF levels after a behaviorally oriented, nutritional rehabilitation program. The subjects underwent BMI, psychomotor speed, and depressive symptom assessments. Statistically significant improvements were found for BMI and depressive symptoms. However, depressive symptoms decrease did not correlate with an increase in BDNF level.

In a naturalistic study by Orosz et al. [54], 71 patients were treated for depression, either single episode or recurrent, with comorbid burnout syndrome. A six-week therapeutic program was followed that involved individual CBT therapy, group therapy, and other therapeutic methods, most notably physical exercise in various forms. Increases in BDNF serum levels were observed, as well as improvements in the Beck Depression Scale, the Insomnia Severity Score, and two out of three scales of the Maslach Burnout Inventory (MBI), i.e., depersonalization and emotional exhaustion. However, the third scale of MBI, i.e., personal efficacy, increased. Heart rate variability was measured to investigate the parasympathetic activity; however, no significant change was observed.

The remaining five studies did not report any statistically significant change in BDNF levels after therapeutic intervention.

In a study by Koch et al. [45] among major depressive disorder patients (*N* = 30) undergoing 12 sessions of interpersonal psychotherapy, there was neither a difference in plasma BDNF levels between baseline and the 21st day of treatment, nor a difference between nonresponder and responder groups, where a response was defined as a reduction of at least 50% of the baseline Hamilton Depression Rating Scale.

Another study by da Silva et al. [52] that analyzed MDD patients (*N* = 55) treated with CBT did not find a change in serum BDNF levels.

In the case of PTSD patients (*N* = 9) treated with prolonged exposure therapy, Powers et al. [49] observed no statistically significant change in plasma BDNF levels [49].

Perroud et al. [47] analyzed a relatively large sample of female BPD patients (*N* = 115), who underwent a four-week DBT program. There was a significant decrease in BDNF protein levels over time, which was inversely associated with treatment response. Responders (>50% improvement in the Beck Depression Scale, BDI) had a nonsignificant increase in BDNF protein levels, while poor responders (≤50% improvement in BDI) showed a nonsignificant decrease in protein levels.

In a study by Rusch et al. [50], 44 soldiers with insomnia underwent CBT-i. Although they observed improvements on clinical scales, there was no significant change in plasma BDNF levels.

Bruijniks et al. [53] analyzed changes in serum BDNF concentration levels in a randomized trial that investigated the effects of session frequency on outcomes in cognitive behavioral therapy and interpersonal psychotherapy for depression. They found no change in BDNF levels after psychotherapy, regardless of therapy type or session frequency. However, this was the only study in which subsequent measurements were conducted after 6 months rather than directly after therapy, which could have influenced BDNF concentration levels.

### 3.2. BDNF Promoter Gene Methylation

The results of two studies that involved BDNF gene methylation status were consistent; both studies examined female borderline patients undergoing dialectical-behavioral therapy. Perroud et al. [47] extracted DNA from peripheral blood leukocytes and investigated the mean methylation in CpG islands located in exon 1 and 4. There was a significant positive association between depression severity, hopelessness, impulsivity, and BDNF methylation status at baseline. Counterintuitively, BDNF methylation significantly increased over time, mainly due to poor responders. After adjusting for baseline clinical scores, the methylation status increased significantly in the nonresponder group and decreased significantly in the responder group. Thus, positive response psychotherapy was linked to a reduction in the mechanisms hampering BDNF gene transcription. In addition, a decrease in depression severity, hopelessness, and impulsivity was observed after treatment and it was significantly and positively associated with methylation status change. Thomas et al. [51] extracted DNA from whole blood and saliva of BPD patients and methylation of four CpG sites in the BDNF promoter was analyzed. They observed a statistically significant decrease in general methylation status in one of four of the analyzed CpG sites in saliva (*N* = 26). However, it did not correlate with a change in clinical symptoms, neither BPD-specific nor general; methylation in the blood samples (*N* = 23) was not altered as compared with the baseline.

## 4. Discussion

In this systematic review, we aimed to evaluate the influence of psychotherapy on peripheral levels of BDNF protein and BDNF gene promoter methylation. The available evidence on the association of psychotherapy and change in BDNF protein levels in plasma or blood serum is inconsistent. In most cases, studies have shown that there were no statistically significant changes in the BDNF concentration levels; only the studies by Orosz et al. [54], Yamada et al. [46], and Zwipp et al. [48] proved otherwise. However, in the case of AN patients, BDNF increase was linked to body weight gain. On the one hand, previous studies have shown that underweight AN patients tended to have lower BDNF concentration levels than those who recovered [55]. Therefore, the observed increase may be due to weight gain, not psychotherapy. On the other hand, Yamada found an increase in BDNF concentration with co-links to weight normalization in BN patients, but the group was small. In the study by Orosz et al. [54], physical exercise was an integrated element of the therapeutic program. Aerobic physical activity is known to positively influence BDNF concentration levels, and therefore it is an important confounding factor which, in this case, could not be controlled [56]. Hence, it is impossible to draw solid conclusions based on this study. The remaining six studies reported no significant changes in BDNF concentration levels. The main limitations of the analyzed studies are the lack of control groups and small experimental groups. In addition, there are a few essential factors that influence BDNF levels, which should be controlled. As previously mentioned, physical activity [41], pharmacotherapy [15,31,32,33,34], and meditation [40] positively influence BDNF concentration levels. A recent meta-analysis showed that ingestion of some polyphenol-rich supplements containing curcumin and *Laminaria japonica* may also have similar effects [57]. A summary of these essential factors and their inclusion in the reviewed articles can be found in Table 2.

There is an expanding body of evidence that effective pharmacological treatment in MDD increases levels of circulating BDNF [31,32,33,58] in the hippocampus, in post mortem studies [59], at least in the case of serotonin selective reuptake inhibitors [34]. However, a recent meta-analysis showed that the importance of peripheral BDNF concentration levels may be less prominent than previously thought [60]. It is also possible that antidepressant action is mediated by stabilizing the BDNF receptor tropomyosin receptor kinase B, rather than increasing the expression of BDNF protein itself [36], which could explain the inconsistency, at least in the case of MDD, and shows different pathways for psychotherapy-induced changes in the brain. In the case of bipolar disorder, BDNF concentration levels are reduced both in manic and depressive states, while, in euthymia, they do not differ as compared with controls [61]. There is also direct proof that successful treatment of manic episode is linked to BDNF level normalization [62]. In the case of schizophrenia, diminished BDNF concentration levels were observed [15,63], as well as their normalization after antipsychotic medication [15]. In addition, non-pharmacological methods also positively influenced BDNF concentration levels in this group of patients [64]. Unexpectedly, only non-exercise methods, such as auditory training and supplementation of probiotics or l-theanine showed a significant effect.

Our literature review is also incongruent with a recent systematic review of psychotherapy influence on BDNF expression. Claudino et al. [44] stated that there was initial evidence for the role of BDNF as an individual psychotherapy response biomarker [44]. However, they counted statistically insignificant changes as important. They also considered the only randomized trial on this topic by Yan et al. [65], which indeed could have been a valuable addition to this body of evidence; however, the aforementioned article was retracted. Concerns were raised relating to the availability and the approved status of the drug (vortioxetine) at the time of the study in the authors’ country [66], and therefore it was excluded from our analysis.

To the best of our knowledge, this is the first systematic review addressing the influence of psychotherapy on BDNF gene methylation status. The two studies included in the review found a decrease in methylation in BPD patients. The study by Perroud et al. [47] showed a difference in blood, while the study by Thomas et al. [51] showed a difference in saliva. The latter also investigated blood samples, but did not reveal a significant difference in methylation, neither at baseline as compared with a control group, nor in a pre-post comparison. This may be explained by methodological differences in the process of determining methylation. Perroud et al. [47] used a high-resolution melting analysis [47]. In contrast, Thomas et al. used pyrosequencing [51]. The main limitation in both studies was the lack of a control group for pre-post comparisons. The results were in line with the results of other studies showing an epigenetic modification of monoamine oxidase A [67] and serotonin transporter [68] genes in the course of psychotherapy. Ziegler et al. [67] investigated patients with panic disorder who were treated with six weekly sessions of exposure-based CBT [67], while Roberts et al. [68] investigated children with different anxiety disorders treated with ten weekly sessions of CBT-based therapy delivered to both children and parents [68]. It is especially interesting that both of the aforementioned studies reported positive changes in responders and negative changes in nonresponders. The results of Perroud et al. [47] are similar [47], which may be a cue that unsuccessful therapy may have harmful consequences at a neurobiological level.

## 5. Conclusions

Taken into consideration all the studies in this review, a few studies reported that peripheral BDNF concentration levels were increased after psychotherapy, while many other studies failed to show a consistent relationship. Nonetheless, methodological issues in the included studies have made drawing solid conclusions difficult. We also underline the necessity to control, in future studies, the aforementioned factors which influence BDNF concentration levels. In addition, the studies on methylation status alterations in the course of psychotherapy, although scarce, were more consistent. It seems to be a more promising direction for future investigation. In both cases, further research with more robust methodology and inclusion of other evidence-based psychotherapy methods is needed.

## Figures and Tables

**Table 1:** Data summary.

Study	Diagnosis	Subjects (N)	Control (N)	Type of Therapy	Length of Therapy	Material Analyzed	Main Findings
Koch et al. (2009) [45]	MDD	30	N/A	IPT ^1^	12 sessions biweekly	Plasma BDNF	No difference
Yamada et al. (2012) [46]	BN	7	N/A	Behavioral program and cognitive treatment	4 weeks	Plasma BDNF	Increased BDNF level without link to BMI or psychological factors, decrease frequency of binge eating and purging behaviors
Perroud et al. (2013) [47]	BPD	115	N/A	DBT ^2^	4 weeks	Serum BDNF/blood BDNF promoter methylation	Nonsignificant increase in BDNF serum levels in therapy responders
Zwipp et al. (2014) [48]	AN	14	N/A	behaviorally oriented, nutritional rehabilitation program	unknown	Serum BDNF	Increased BDNF level after weight gain
Powers et al. (2015) [49]	PTSD	9	N/A	Prolonged exposure therapy	12 sessions	Plasma BDNF	No difference
Rusch et al. (2015) [50]	Insomnia	44	N/A	CBT-I ^3^	4–8 sessions	Plasma BDNF	Nonsignificant BDNF increase in responders, significant reductions in depression, posttraumatic arousal symptoms, improvement in emotional well-being and energy/fatigue in responders
Thomas et al. (2018) [51]	BPD	26	N/A	DBT	12 weeks	Blood/Saliva BDNF promoter methylation	Decrease in BDNF IV promoter methylation, no differences in methylation change between patients with and without significant improvement
Da Silva et al. (2018) [52]	MDD	55	N/A	CBT ^4^	16 sessions	Serum BDNF	No difference
Bruijniks et al. (2020) [53]	MDD	82	N/A	IPT/CBT	once or twice a week for 16–24 weeks, maximum 20 sessions	Serum	No difference
Orosz et al. (2020) [54]	MMD, burnout	71	N/A	Individual CBT Group therapy	6 weeks	Serum BDNF	Increase in BDNF levels, improvement in sleep, depressive symptoms, emotional exhaustion, and depersonalization, but not personal efficacy and parasympathetic activity

^1^, interpersonal therapy; ^2^, dialectical behavioral therapy; ^3^, cognitive behavioral therapy for insomnia; ^4^, cognitive behavioral therapy.

**Table 2:** Factors influencing peripheral BDNF concentration levels controlled in the studies included in the review.

Study	Age	Sex	Physical Activity	Meditation	Pharmacotherapy	Supplements	Comorbidity
Koch et al. (2009) [45]	+	+	−	−	+	−	+ *
Yamada et al. (2012) [46]	+	+	−	−	+	−	+
Perroud et al. (2013) [47]	+	+	−	−	+	−	−
Zwipp et al. (2014) [48]	+	+	−	−	+	−	+
Powers et al. (2015) [49]	−	−	+	−	+	−	+
Rusch et al. (2015) [50]	+	+	−	−	+	−	+
Da Silva et al. (2018) [52]	+	+	−	−	+	−	+
Bruijniks et al. (2020) [53]	+	+	−	−	+	−	+
Orosz et al. (2020) [54]	+	+	−	−	+	−	+ *

+, included; −, not included; n/a, not applicable; *, not including important neurological conditions.

## References

[1] Weingarten C.P., Strauman T.J. (2015). Neuroimaging for psychotherapy research: Current trends. Psychother. Res..

[2] Binder D.K., Scharfman H.E. (2004). Brain-derived neurotrophic factor. Growth Factors.

[3] Shimada H., Makizako H., Doi T., Yoshida D., Tsutsumimoto K., Anan Y., Uemura K., Lee S., Park H., Suzuki T. (2014). A large, cross-sectional observational study of serum BDNF, cognitive function, and mild cognitive impairment in the elderly. Front. Aging Neurosci..

[4] Miranda M., Morici J.F., Zanoni M.B., Bekinschtein P. (2019). Brain-Derived Neurotrophic Factor: A Key Molecule for Memory in the Healthy and the Pathological Brain. Front. Cell. Neurosci..

[5] Mizoguchi Y., Yao H., Imamura Y., Hashimoto M., Monji A. (2020). Lower brain-derived neurotrophic factor levels are associated with age-related memory impairment in community-dwelling older adults: The Sefuri study. Sci. Rep..

[6] Hariri A.R., Goldberg T.E., Mattay V.S., Kolachana B.S., Callicott J.H., Egan M.F., Weinberger D.R. (2003). Brain-derived neurotrophic factor val66met polymorphism affects human memory-related hippocampal activity and predicts memory performance. J. Neurosci. Off. J. Soc. Neurosci..

[7] Goldberg T.E., Iudicello J., Russo C., Elvevåg B., Straub R., Egan M.F., Weinberger D.R. (2008). BDNF Val66Met polymorphism significantly affects d’ in verbal recognition memory at short and long delays. Biol. Psychol..

[8] Egan M.F., Kojima M., Callicott J.H., Goldberg T.E., Kolachana B.S., Bertolino A., Zaitsev E., Gold B., Goldman D., Dean M. (2003). The BDNF val66met Polymorphism Affects Activity-Dependent Secretion of BDNF and Human Memory and Hippocampal Function. Cell.

[9] Autry A.E., Monteggia L.M. (2012). Brain-derived neurotrophic factor and neuropsychiatric disorders. Pharm. Rev..

[10] Youssef M.M., Underwood M.D., Huang Y.-Y., Hsiung S.-C., Liu Y., Simpson N.R., Bakalian M.J., Rosoklija G.B., Dwork A.J., Arango V. (2018). Association of BDNF Val66Met Polymorphism and Brain BDNF Levels with Major Depression and Suicide. Int. J. Neuropsychopharmacol..

[11] Sklar P., Gabriel S.B., McInnis M.G., Bennett P., Lim Y., Tsan G., Schaffner S., Kirov G., Jones I., Owen M. (2002). Family-based association study of 76 candidate genes in bipolar disorder: BDNF is a potential risk locus. Brain-derived neutrophic factor. Mol. Psychiatry.

[12] Sarchiapone M., Carli V., Roy A., Iacoviello L., Cuomo C., Latella M.C., di Giannantonio M., Janiri L., de Gaetano M., Janal M.N. (2008). Association of polymorphism (Val66Met) of brain-derived neurotrophic factor with suicide attempts in depressed patients. Neuropsychobiology.

[13] Brandys M.K., Kas M.J., van Elburg A.A., Campbell I.C., Adan R.A. (2011). A meta-analysis of circulating BDNF concentrations in anorexia nervosa. World J. Biol. Psychiatry.

[14] Sen S., Duman R., Sanacora G. (2008). Serum brain-derived neurotrophic factor, depression, and antidepressant medications: Meta-analyses and implications. Biol. Psychiatry.

[15] Fernandes B.S., Steiner J., Berk M., Molendijk M.L., Gonzalez-Pinto A., Turck C.W., Nardin P., Gonçalves C.A. (2015). Peripheral brain-derived neurotrophic factor in schizophrenia and the role of antipsychotics: Meta-analysis and implications. Mol. Psychiatry.

[16] Ng T.K.S., Ho C.S.H., Tam W.W.S., Kua E.H., Ho R.C.-M. (2019). Decreased Serum Brain-Derived Neurotrophic Factor (BDNF) Levels in Patients with Alzheimer’s Disease (AD): A Systematic Review and Meta-Analysis. Int. J. Mol. Sci..

[17] Iughetti L., Lucaccioni L., Fugetto F., Predieri B., Berardi A., Ferrari F. (2018). Brain-derived neurotrophic factor and epilepsy: A systematic review. Neuropeptides.

[18] Roth T.L., Sweatt J.D. (2011). Epigenetic marking of the BDNF gene by early-life adverse experiences. Horm. Behav..

[19] Miao Z., Wang Y., Sun Z. (2020). The Relationships between Stress, Mental Disorders, and Epigenetic Regulation of BDNF. Int. J. Mol. Sci..

[20] Moser D.A., Paoloni-Giacobino A., Stenz L., Adouan W., Manini A., Suardi F., Cordero M.I., Vital M., Sancho Rossignol A., Rusconi-Serpa S. (2015). BDNF Methylation and Maternal Brain Activity in a Violence-Related Sample. PLoS ONE.

[21] Thaler L., Gauvin L., Joober R., Groleau P., de Guzman R., Ambalavanan A., Israel M., Wilson S., Steiger H. (2014). Methylation of BDNF in women with bulimic eating syndromes: Associations with childhood abuse and borderline personality disorder. Prog. Neuropsychopharmacol. Biol. Psychiatry.

[22] Kim T.Y., Kim S.J., Chung H.G., Choi J.H., Kim S.H., Kang J.I. (2017). Epigenetic alterations of the BDNF gene in combat-related post-traumatic stress disorder. Acta Psychiatr. Scand..

[23] Miller J.K., McDougall S., Thomas S., Wiener J. (2017). The Impact of the Brain-Derived Neurotrophic Factor Gene on Trauma and Spatial Processing. J. Clin. Med..

[24] Wu G.W.Y., Wolkowitz O.M., Reus V.I., Kang J.I., Elnar M., Sarwal R., Flory J.D., Abu-Amara D., Hammanieh R., Gautam A. (2021). Serum brain-derived neurotrophic factor remains elevated after long term follow-up of combat veterans with chronic post-traumatic stress disorder. Psychoneuroendocrinology.

[25] Hauck S., Kapczinski F., Roesler R., de Moura Silveira E., Magalhães P.V., Kruel L.R., Schestatsky S.S., Ceitlin L.H. (2010). Serum brain-derived neurotrophic factor in patients with trauma psychopathology. Prog. Neuropsychopharmacol. Biol. Psychiatry.

[26] Matsuoka Y., Nishi D., Noguchi H., Kim Y., Hashimoto K. (2013). Longitudinal changes in serum brain-derived neurotrophic factor in accident survivors with posttraumatic stress disorder. Neuropsychobiology.

[27] Dell’Osso L., Carmassi C., Del Debbio A., Catena Dell’Osso M., Bianchi C., da Pozzo E., Origlia N., Domenici L., Massimetti G., Marazziti D. (2009). Brain-derived neurotrophic factor plasma levels in patients suffering from post-traumatic stress disorder. Prog. Neuropsychopharmacol. Biol. Psychiatry.

[28] Angelucci F., Ricci V., Gelfo F., Martinotti G., Brunetti M., Sepede G., Signorelli M., Aguglia E., Pettorruso M., Vellante F. (2014). BDNF serum levels in subjects developing or not post-traumatic stress disorder after trauma exposure. Brain Cogn..

[29] Toledo-Rodriguez M., Lotfipour S., Leonard G., Perron M., Richer L., Veillette S., Pausova Z., Paus T. (2010). Maternal smoking during pregnancy is associated with epigenetic modifications of the brain-derived neurotrophic factor-6 exon in adolescent offspring. Am. J. Med. Genet. B Neuropsychiatr. Genet..

[30] Zheleznyakova G.Y., Cao H., Schiöth H.B. (2016). BDNF DNA methylation changes as a biomarker of psychiatric disorders: Literature review and open access database analysis. Behav. Brain Funct..

[31] Matrisciano F., Bonaccorso S., Ricciardi A., Scaccianoce S., Panaccione I., Wang L., Ruberto A., Tatarelli R., Nicoletti F., Girardi P. (2009). Changes in BDNF serum levels in patients with major depression disorder (MDD) after 6 months treatment with sertraline, escitalopram, or venlafaxine. J. Psychiatr. Res.

[32] Yoshimura R., Mitoma M., Sugita A., Hori H., Okamoto T., Umene W., Ueda N., Nakamura J. (2007). Effects of paroxetine or milnacipran on serum brain-derived neurotrophic factor in depressed patients. Prog. Neuropsychopharmacol. Biol. Psychiatry.

[33] Hellweg R., Ziegenhorn A., Heuser I., Deuschle M. (2008). Serum concentrations of nerve growth factor and brain-derived neurotrophic factor in depressed patients before and after antidepressant treatment. Pharmacopsychiatry.

[34] Molendijk M.L., Bus B.A., Spinhoven P., Penninx B.W., Kenis G., Prickaerts J., Voshaar R.C., Elzinga B.M. (2011). Serum levels of brain-derived neurotrophic factor in major depressive disorder: State-trait issues, clinical features and pharmacological treatment. Mol. Psychiatry.

[35] Liu X., Zhang J., Sun D., Fan Y., Zhou H., Fu B. (2014). Effects of fluoxetine on brain-derived neurotrophic factor serum concentration and cognition in patients with vascular dementia. Clin. Interv. Aging.

[36] Castrén E., Monteggia L.M. (2021). Brain-Derived Neurotrophic Factor Signaling in Depression and Antidepressant Action. Biol. Psychiatry.

[37] Zheng F., Wang H. (2009). NMDA-mediated and self-induced bdnf exon IV transcriptions are differentially regulated in cultured cortical neurons. Neurochem. Int..

[38] Vanicek T., Kranz G.S., Vyssoki B., Komorowski A., Fugger G., Höflich A., Micskei Z., Milovic S., Lanzenberger R., Eckert A. (2019). Repetitive enhancement of serum BDNF subsequent to continuation ECT. Acta Psychiatr. Scand..

[39] Rocha R.B., Dondossola E.R., Grande A.J., Colonetti T., Ceretta L.B., Passos I.C., Quevedo J., da Rosa M.I. (2016). Increased BDNF levels after electroconvulsive therapy in patients with major depressive disorder: A meta-analysis study. J. Psychiatr. Res..

[40] Gomutbutra P., Yingchankul N., Chattipakorn N., Chattipakorn S., Srisurapanont M. (2020). The Effect of Mindfulness-Based Intervention on Brain-Derived Neurotrophic Factor (BDNF): A Systematic Review and Meta-Analysis of Controlled Trials. Front. Psychol..

[41] Szuhany K.L., Bugatti M., Otto M.W. (2015). A meta-analytic review of the effects of exercise on brain-derived neurotrophic factor. J. Psychiatr. Res..

[42] Kim K., Sung Y.-H., Seo J.-H., Lee S.-W., Lim B.-V., Lee C.-Y., Chung Y.-R. (2015). Effects of treadmill exercise-intensity on short-term memory in the rats born of the lipopolysaccharide-exposed maternal rats. J. Exerc. Rehabil..

[43] Gomez-Pinilla F., Zhuang Y., Feng J., Ying Z., Fan G. (2011). Exercise impacts brain-derived neurotrophic factor plasticity by engaging mechanisms of epigenetic regulation. Eur. J. Neurosci..

[44] Claudino F.C.A., Gonçalves L., Schuch F.B., Martins H.R.S., da Rocha N.S. (2020). The Effects of Individual Psychotherapy in BDNF Levels of Patients with Mental Disorders: A Systematic Review. Front. Psychiatry.

[45] Koch J.M., Hinze-Selch D., Stingele K., Huchzermeier C., Goder R., Seeck-Hirschner M., Aldenhoff J.B. (2009). Changes in CREB Phosphorylation and BDNF Plasma Levels during Psychotherapy of Depression. Psychother. Psychosom..

[46] Yamada H., Yoshimura C., Nakajima T., Nagata T. (2012). Recovery of low plasma BDNF over the course of treatment among patients with bulimia nervosa. Psychiatry Res..

[47] Perroud N., Salzmann A., Prada P., Nicastro R., Hoeppli M.E., Furrer S., Ardu S., Krejci I., Karege F., Malafosse A. (2013). Response to psychotherapy in borderline personality disorder and methylation status of the BDNF gene. Transl. Psychiatry.

[48] Zwipp J., Hass J., Schober I., Geisler D., Ritschel F., Seidel M., Weiss J., Roessner V., Hellweg R., Ehrlich S. (2014). Serum brain-derived neurotrophic factor and cognitive functioning in underweight, weight-recovered and partially weight-recovered females with anorexia nervosa. Prog. Neuropsychopharmacol. Biol. Psychiatry.

[49] Powers M.B., Medina J.L., Burns S., Kauffman B.Y., Monfils M., Asmundson G.J., Diamond A., McIntyre C., Smits J.A. (2015). Exercise Augmentation of Exposure Therapy for PTSD: Rationale and Pilot Efficacy Data. Cogn. Behav. Ther..

[50] Rusch H.L., Guardado P., Baxter T., Mysliwiec V., Gill J.M. (2015). Improved Sleep Quality is Associated with Reductions in Depression and PTSD Arousal Symptoms and Increases in IGF-1 Concentrations. J. Clin. Sleep Med..

[51] Thomas M., Knoblich N., Wallisch A., Glowacz K., Becker-Sadzio J., Gundel F., Bruckmann C., Nieratschker V. (2018). Increased BDNF methylation in saliva, but not blood, of patients with borderline personality disorder. Clin. Epigenet..

[52] da Silva S.K., Wiener C., Ghisleni G., Oses J.P., Jansen K., Molina M.L., Silva R., Souza L.D. (2018). Effects of cognitive-behavioral therapy on neurotrophic factors in patients with major depressive disorder. Rev. Bras. Psiquiatr..

[53] Bruijniks S.J.E., van Grootheest G., Cuijpers P., de Kluiver H., Vinkers C.H., Peeters F., Penninx B., Teunissen C.E., Huibers M.J.H. (2020). Working memory moderates the relation between the brain-derived neurotropic factor (BDNF) and psychotherapy outcome for depression. J. Psychiatr. Res..

[54] Orosz A., Federspiel A., Eckert A., Seeher C., Dierks T., Tschitsaz A., Cattapan K. (2021). Exploring the effectiveness of a specialized therapy programme for burnout using subjective report and biomarkers of stress. Clin. Psychol. Psychother..

[55] Ehrlich S., Salbach-Andrae H., Eckart S., Merle J.V., Burghardt R., Pfeiffer E., Franke L., Uebelhack R., Lehmkuhl U., Hellweg R. (2009). Serum brain-derived neurotrophic factor and peripheral indicators of the serotonin system in underweight and weight-recovered adolescent girls and women with anorexia nervosa. J. Psychiatry Neurosci..

[56] Phillips C. (2017). Brain-Derived Neurotrophic Factor, Depression, and Physical Activity: Making the Neuroplastic Connection. Neural Plast..

[57] Gravesteijn E., Mensink R.P., Plat J. (2021). Effects of nutritional interventions on BDNF concentrations in humans: A systematic review. Nutr. Neurosci..

[58] Lopez J.P., Mamdani F., Labonte B., Beaulieu M.M., Yang J.P., Berlim M.T., Ernst C., Turecki G. (2013). Epigenetic regulation of BDNF expression according to antidepressant response. Mol. Psychiatry.

[59] Chen B., Dowlatshahi D., MacQueen G.M., Wang J.F., Young L.T. (2001). Increased hippocampal BDNF immunoreactivity in subjects treated with antidepressant medication. Biol. Psychiatry.

[60] Molendijk M.L., Spinhoven P., Polak M., Bus B.A., Penninx B.W., Elzinga B.M. (2014). Serum BDNF concentrations as peripheral manifestations of depression: Evidence from a systematic review and meta-analyses on 179 associations (N = 9484). Mol. Psychiatry.

[61] Rowland T., Perry B.I., Upthegrove R., Barnes N., Chatterjee J., Gallacher D., Marwaha S. (2018). Neurotrophins, cytokines, oxidative stress mediators and mood state in bipolar disorder: Systematic review and meta-analyses. Br. J. Psychiatry J. Ment. Sci..

[62] Fernandes B.S., Molendijk M.L., Köhler C.A., Soares J.C., Leite C.M.G.S., Machado-Vieira R., Ribeiro T.L., Silva J.C., Sales P.M.G., Quevedo J. (2015). Peripheral brain-derived neurotrophic factor (BDNF) as a biomarker in bipolar disorder: A meta-analysis of 52 studies. BMC Med..

[63] Çakici N., Sutterland A.L., Penninx B., Dalm V.A., de Haan L., van Beveren N.J.M. (2020). Altered peripheral blood compounds in drug-naïve first-episode patients with either schizophrenia or major depressive disorder: A meta-analysis. Brain Behav. Immun..

[64] Sanada K., Zorrilla I., Iwata Y., Bermúdez-Ampudia C., Graff-Guerrero A., Martínez-Cengotitabengoa M., González-Pinto A. (2016). The Efficacy of Non-Pharmacological Interventions on Brain-Derived Neurotrophic Factor in Schizophrenia: A Systematic Review and Meta-Analysis. Int. J. Mol. Sci..

[65] Yan G., Zhang M., Liu Y., Yin M. (2019). Efficacy of vortioxetine combined cognitive behaviour intervention therapy on brain-derived neurotrophic factor level on depressive patients. Psychogeriatrics.

[66] (2021). Retraction. Psychogeriatrics.

[67] Ziegler C., Richter J., Mahr M., Gajewska A., Schiele M.A., Gehrmann A., Schmidt B., Lesch K.P., Lang T., Helbig-Lang S. (2016). MAOA gene hypomethylation in panic disorder-reversibility of an epigenetic risk pattern by psychotherapy. Transl. Psychiatry.

[68] Roberts S., Lester K.J., Hudson J.L., Rapee R.M., Creswell C., Cooper P.J., Thirlwall K.J., Coleman J.R., Breen G., Wong C.C. (2014). Serotonin transporter [corrected] methylation and response to cognitive behaviour therapy in children with anxiety disorders. Transl. Psychiatry.

